# Chronic Iliac Vein Occlusion and Painful Nonhealing Ulcer Induced by High Venous Pressures from an Arteriovenous Malformation

**DOI:** 10.1155/2011/514721

**Published:** 2011-12-12

**Authors:** Daniel P. Link, Phillip J. Granchi

**Affiliations:** University of California Davis, School of Medicine, 4860 Y Street, Suite 3400, Sacramento, CA 96817, USA

## Abstract

Chronic femoral vein compression (May-Thurner Syndrome) is a known rare cause of deep venous thrombosis. Subsequent angiogenesis and the development of arteriovenous malformation (AVM) in the setting of chronic venous thrombosis is by itself a rare and poorly understood phenomenon. We report a case in which elevated venous pressures resulting from such compression appear to have resulted in the development of a pelvic arteriovenous malformation, which was further complicated by chronic, nonhealing painful lower extremity ulcers, and the development of extensive subcutaneous venous collaterals. Following successful embolization of the pelvic AVM and ablation of veins under the ulcers with laser and sclerotherapy, the patient's ulcers healed and she became pain-free.

## 1. Introduction

Venous thrombosis is an established inciting factor of angiogenesis in both humans and animals (Varnagy). Previously published case reports have shown that peripheral AVMs may develop as a result of such angiogenesis with a hypothesized “vascular stress” mechanism of control (Link/Monsky/Garza). The development of high extremity venous pressures that result from and a thrombosis-induced AVM may lead to painful and difficult to manage ulcerations of the skin.

## 2. Case Report

**Figure 1:**
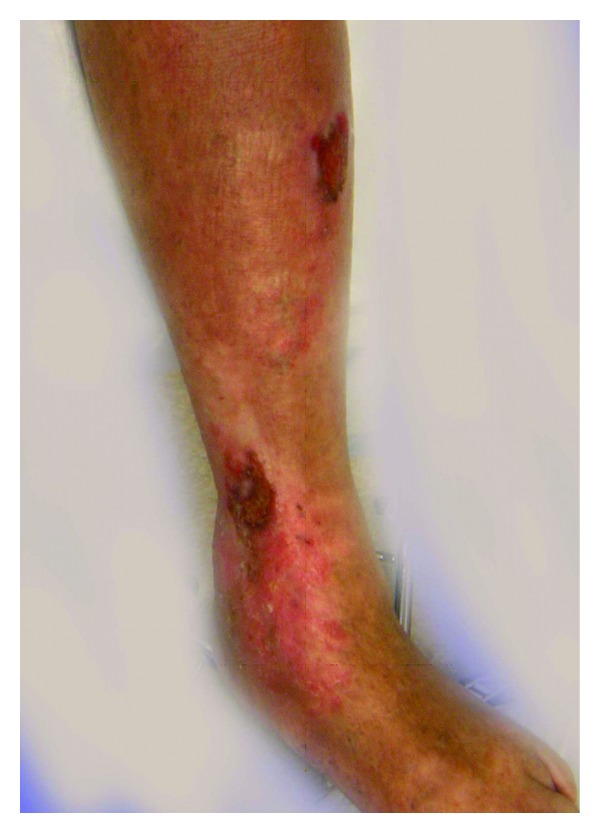
Large nonhealing leg ulcers.

**Figure 2:**
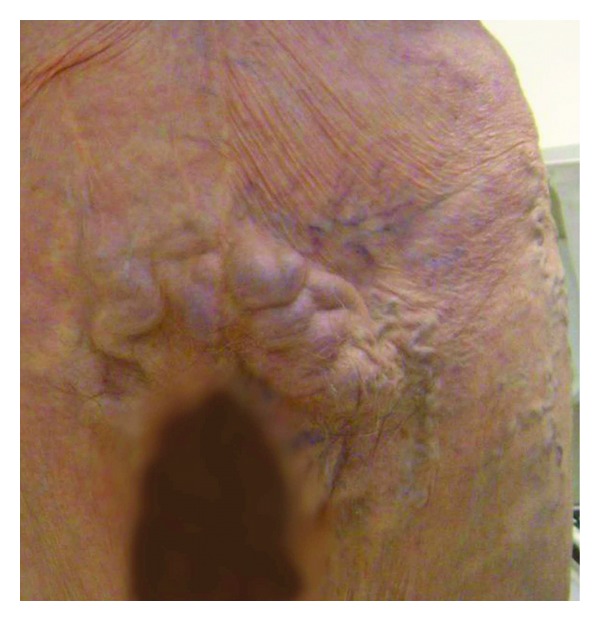
Large subcutaneous collateral veins.

**Figure 3:**
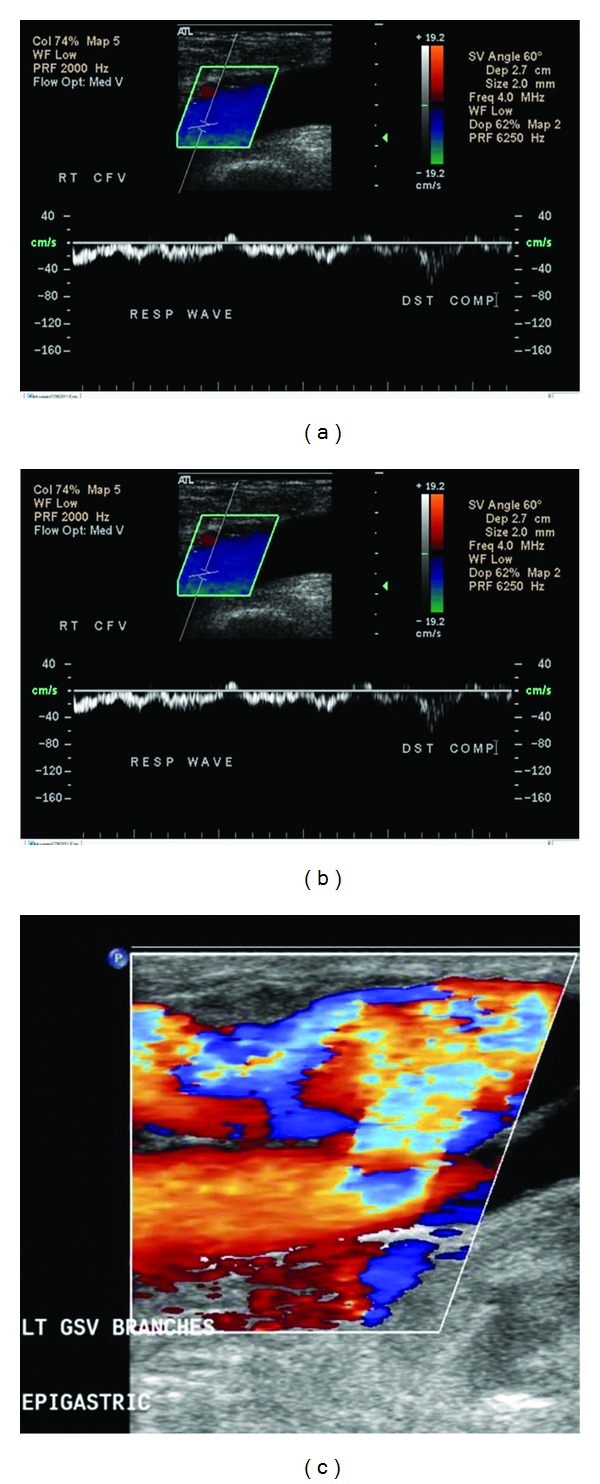
(a) Normal-to-high signal in the left common femoral vein 3 years before presentation. (b) Increased Doppler velocity in the left common femoral vein 1 year prior to presentation. (c) Marked turbulence and high flow at the junction of the left great saphenous vein with the femoral vein.

**Figure 4:**
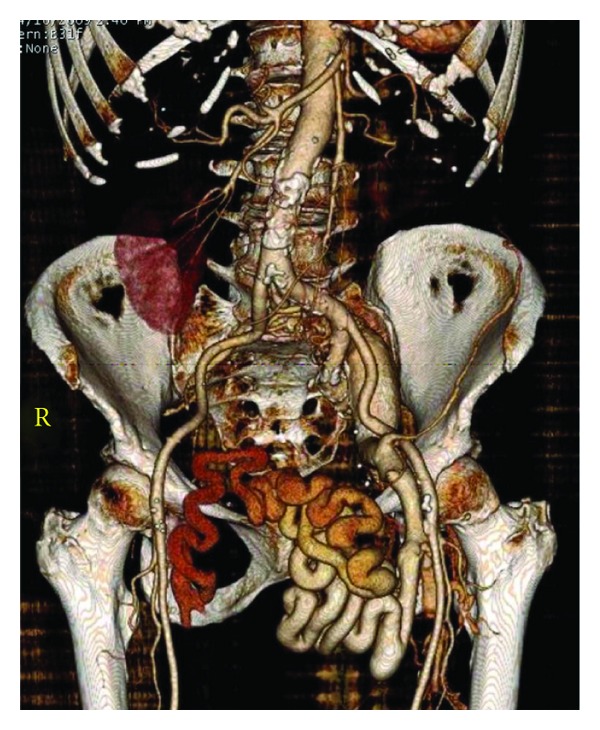
3D reconstruction of the left pelvic AVM with outflow to the left external iliac vein and collateral veins leading to the right common femoral vein (not shown). The supplying arteries are the left internal iliac a. (arrow) and the left 5th lumbar a. (arrowhead).

**Figure 5:**
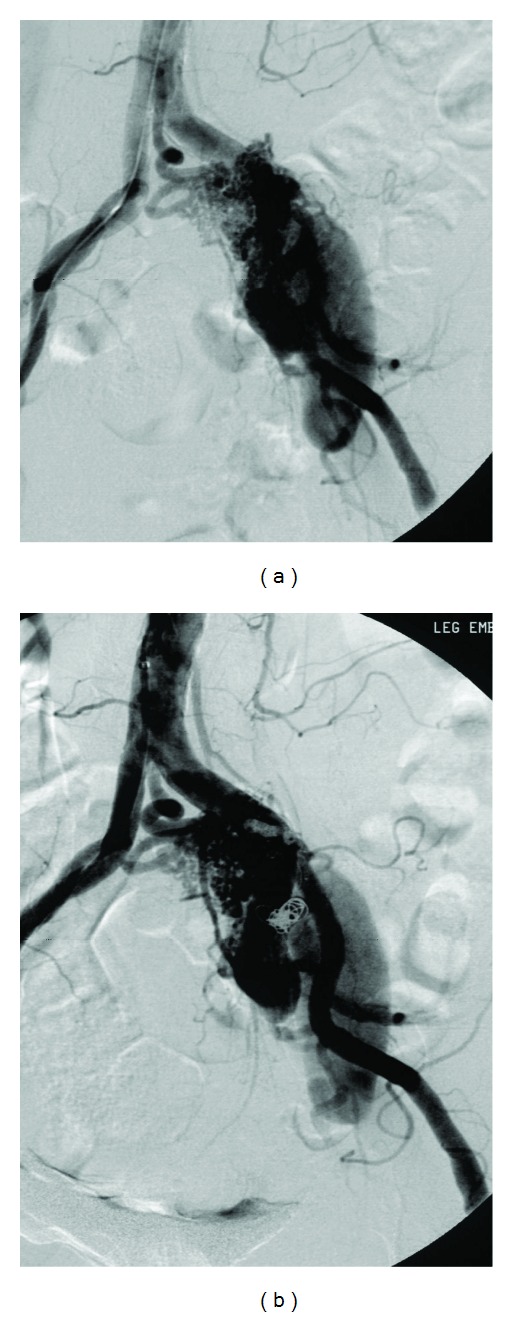
(a) Arteriogram of the AVM, preembolization, left 5th lumbar artery (arrow head) and the left internal iliac artery (arrow) are the major feeding vessels. (b) Postembolization with Onyx (EV3, Irvine, CA, USA) and coils (Cook Inc).

**Figure 6:**
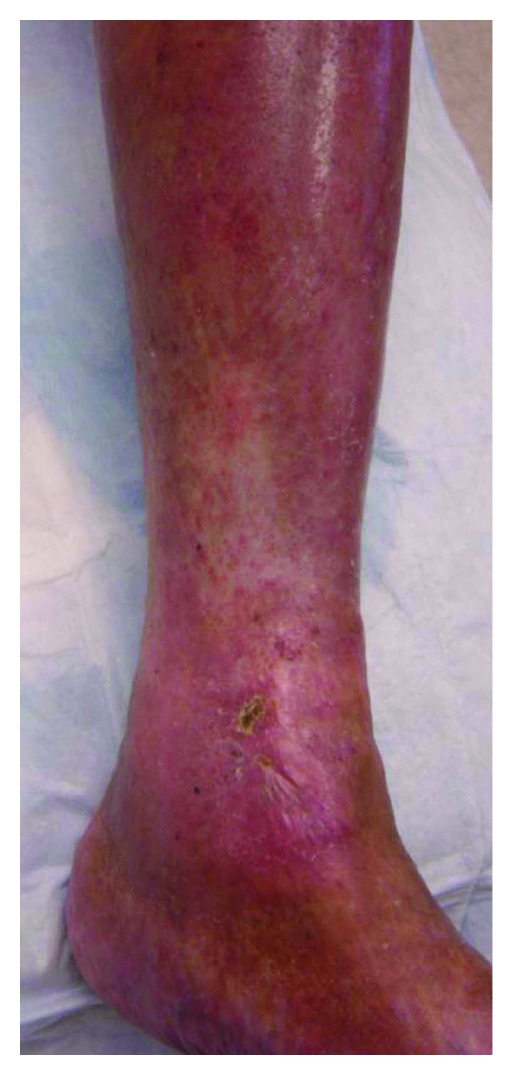
Healing of the skin ulcers following embolization, endovenous ablation, and sclerotherapy.

An 86-year-old woman first presented to the Vascular Center Clinic with a chronic nonhealing, painful ulcers over the medial malleolus and anterior left shin, Figure 1. She had been seen in various clinics for the last 7 years during which time the ulcers progressed and the pain intensified despite compressive dressings [1]. By history, the patient, previously healthy, suffered a left ankle fracture that led to left-sided deep vein thrombosis (DVT). After a short term of anticoagulation, she began to develop the pigmentation that subsequently led to skin ulceration over the medial malleolus. The collateral veins had not been mentioned previously, Figure 2. The duplex studies from previous years had shown a sequential increase in the femoral vein Doppler signal velocity and turbulence, Figures 3(a), 3(b), and 3(c). A CTA showed an AVM with supplying arteries from the left internal iliac a. and the 5th lumbar artery, Figures 4(a) and 4(b). An embolization was performed using Onyx liquid embolic agent (EV3, Irvine, CA, USA) and embolization coils (Cook Inc, Bloomington, IN, USA), Figure 5. She had partial relief of her pain and some healing in the ulcer beds. The veins deep to the ulcers were accessed with a 25 gauge needle and injected with 0.5% sotradecol solution (AngioDynamics, Latham, NY, USA), and the largest tributary vein to the ulcer bed as determined by duplex examination in the clinic was treated with a 400 micron laser fiber (AngioDynamics Latham, NY, USA). Compression bandages for another few weeks led to healing of the ulcers and complete resolution of the pain, Figure 6. 

## 3. Discussion

This case represents a rare late sequela of venous thrombosis that can be effectively treated when recognized. The predominance for left-sided venous thrombosis has been recognized [2, 3]. These authors who dissected cadavers reported anomalies of the iliac v. that were associated with thrombosis. Thrombus contains an environment that leads to revascularization and may in this process lead to lesions that rapidly shunt arterial flow to the venous system, AVM. Such shunting has been described in the transverse sinuses [4, 5] and in the iliac and femoral veins [6]. The findings are interesting in that the collateral arteries supplying the AVM probably represent the source for the vasa vasorum of the left internal iliac vein as this portion of the venous system develops from the embryonic segments at this level [7]. Studies showing a robust response of the vasa vasorum, including vasculogenesis, to disease states in the arteries have been recognized, [8, 9] and are of interest but a similar response of the venous vasa vasorum could be expected. The development of an AVM or artializatoin of an occluded venous system can be suspected in the DVT patient who continues to have severe pain and progression of skin changes despite adequat compresion therapy. The diagnosis can be confirmed by duplex imaging as in this case.

Treatment of such patients is palliative and involves depressurization of the occluded venous system especially the subepidermal complexes of veins deep to the ulcer. A venous reconstruction is sometimes possible, [10]. This patient's age precluded this procedure or any procedure requiring general anesthesia. [11]. The embolization was performed with sedation only which directed the use of the chosen agents. The marked reduction in the arterialized flow was not enough to heal the ulcer beds. Sclerotherapy for intractable ulcers is being performed in most clinics but has seldom been reported [12]. It can be very effective in the treatment of stasis not responding to standard compression therapy.

## 4. Conclusion

Early recognition of arterialized veins distal to iliac and femoral vein occlusions lead to pain and ulcers that are difficult to treat but can be managed by minimally invasive techniques.
